# Nd^3+^-Doped TiO_2_ Nanoparticles as Nanothermometer: High Sensitivity in Temperature Evaluation inside Biological Windows

**DOI:** 10.3390/s21165306

**Published:** 2021-08-06

**Authors:** Selene Acosta, Luis J. Borrero-González, Polona Umek, Luiz A. O. Nunes, Peter Guttmann, Carla Bittencourt

**Affiliations:** 1 Chimie des Interactions Plasma-Surface (ChIPS), Research Institute for Materials Science and Engineering, Université de Mons, 7000 Mons, Belgium; carla.bittencourt@umons.ac.be; 2 Centro de Investigación en Ciencias de la Salud y Biomedicina, Universidad Autónoma de San Luis Potosí., San Luis Potosí 78210, Mexico; 3 Facultad de Ciencias Exactas y Naturales, Escuela de Ciencias Físicas y Matemática, Pontificia Universidad Católica del Ecuador, Av. 12 de Octubre 1076, Apartado, Quito 17-01-2184, Ecuador; LJBORRERO@puce.edu.ec; 4 Jožef Stefan Institute, Jamova Cesta 39, 1000 Ljubljana, Slovenia; polona.umek@ijs.si; 5 Instituto de Física de São Carlos, Universidade de São Paulo, São Carlos 13566-590, Brazil; luizant@ifsc.usp.br; 6 Helmholtz-Zentrum Berlin für Materialien und Energie GmbH, Department X-Ray Microscopy, Wilhelm-Conrad-Röntgen Campus, 12489 Berlin, Germany; peter.guttmann@helmholtz-berlin.de

**Keywords:** TiO_2_, luminescence, Nd, nanothermometer

## Abstract

TiO_2_ nanoparticles doped with different amounts of Nd^3+^ (0.5, 1, and 3 wt.%) were synthetized by the sol–gel method, and evaluated as potential temperature nanoprobes using the fluorescence intensity ratio between thermal-sensitive radiative transitions of the Nd^3+^. XRD characterization identified the anatase phase in all the doped samples. The morphology of the nanoparticles was observed with SEM, TEM and HRTEM microscopies. The relative amount of Nd^3+^ in TiO_2_ was obtained by EDXS, and the oxidation state of titanium and neodymium was investigated via XPS and NEXAFS, respectively. Nd^3+^ was present in all the samples, unlike titanium, where besides Ti^4+^, a significantly amount of Ti^3+^ was observed; the relative concentration of Ti^3+^ increased as the amount of Nd^3+^ in the TiO_2_ nanoparticles increased. The photoluminescence of the synthetized nanoparticles was investigated, with excitation wavelengths of 350, 514 and 600 nm. The emission intensity of the broad band that was associated with the presence of defects in the TiO_2_, increased when the concentration of Nd^3+^ was increased. Using 600 nm for excitation, the ^4^F_7/2_→^4^I_9/2_, ^4^F_5/2_→^4^I_9/2_ and ^4^F_3/2_→^4^I_9/2_ transitions of Nd^3+^ ions, centered at 760 nm, 821 nm, and 880 nm, respectively, were observed. Finally, the effect of temperature in the photoluminescence intensity of the synthetized nanoparticles was investigated, with an excitation wavelength of 600 nm. The spectra were collected in the 288–348 K range. For increasing temperatures, the emission intensity of the ^4^F_7/2_→^4^I_9/2_ and ^4^F_5/2_→^4^I_9/2_ transitions increased significantly, in contrast to the ^4^F_3/2_→^4^I_9/2_ transition, in which the intensity emission decreased. The fluorescence intensity ratio between the transitions I821I880=F5/24I49/2F43/2I49/2 and I760I880=F47/2I49/2F43/2I49/2 were used to calculate the relative sensitivity of the sensors. The relative sensitivity was near 3% K^−1^ for $\frac{I_{760}}{I_{880}}$ and near 1% K^−1^ for $\frac{I_{821}}{I_{880}}$.

## 1. Introduction

TiO_2_, also called titania, is widely studied, due to its large chemical stability, easy and low-cost synthesis methods, additionally to its high photoactivity [1,2]; these properties make TiO_2_ a good candidate for several valuable applications, such as solar cells, gas sensors, water cleaning, among others [3,4,5,6,7,8]. Furthermore, due to its high biocompatibility, TiO_2_ is a promising material for biological applications [9,10]. The optical properties of TiO_2_ depend on the density and type of defects, the presence of dopants, as well as on the synthesis method [11]. TiO_2_ presents high transparency in visible/NIR light, and absorbs light in the UV region of the spectrum, due to its large band gap (~3.2 eV) [12]; the UV region represents just 3–5% of the solar spectrum [13]. However, it has been shown that doping titania can lead to changes in its optical properties [14,15,16,17]; doping TiO_2_ with lanthanide ions allows the system to be excited with visible light (>400 nm), thus a great part of the solar spectrum can be used to excite Ln^3+^ –TiO_2_ [18]. The emission lines of the different lanthanides appear in all the regions of the spectrum, from UV (Gd^3+^), the visible range (Eu^3+^, Sm^3+^), to the near-infrared (NIR) range (Nd^3+^, Er^3+^, Yb^3+^) [19]. Lanthanide ions owe their unique luminescence properties to electronic transitions in their partially filled *4f* orbitals. The emission lines of lanthanides are very narrow, specific, and with long lifetimes, due to the shielded of *4f* levels by the *5s* and *5p* levels that protect the *f–f* transitions from perturbations caused by the crystalline field of the host matrix. However, the intensity and width of the lines of photoluminescence depend on the host material [20,21]. Bearing in mind the information above, choosing among different lanthanides and host matrices, allows the emission of the material to be adjusted for a desired application. Nevertheless, using lanthanides as dopants with high concentrations produces dopant clustering and luminescence quenching [14,22,23,24], consequently for each host there is a doping limit to avoid clustering, and strategies to detect and analyze low photoluminescence intensity signals must be developed. 

Among the Ln^3+^ ions, Nd^3+^ has obtained particular attention, due to its singular optical and magnetic properties [25,26,27]. It has been reported that the introduction of neodymium atoms into the TiO_2_ lattice induces the formation of new sub-bandgap states below the TiO_2_ conduction band, due to the properly overlap of the Nd^3+^ energy levels with the conduction band of TiO_2_ [12] decreasing the energy of the TiO_2_ bandgap [28]. Additionally, Hassan et al. [29], reported that Nd^3+^_,_ compared with Ce^3+^ and La^3+^ ions, had led to a higher increase in TiO_2_ photoactivity. While Silva et al. used Nd^3+^-doped TiO_2_ nanocrystals as a luminescent nanothermometer operating within biological windows [30].

The increased interest in nanometer scale systems, such as in nanoelectronics and nanofluids, has increased the need to monitor local changes in the temperature in nanometric scales, which traditional thermometers are unable to measure. Different strategies have been developed to tackle this need, one of the most studied is luminescence nanothermometry, which is a technique that uses materials whose luminescent properties, such as luminescence intensity, bandwidth, polarization, decay time, spectral position, and band line shape, are affected by temperature [31]. It is recognized that the luminescent properties of Ln^3+^ ions, such as luminescent intensity, depend critically on the temperature [32,33,34]. Besides this, the relative long recombination lifetimes compared to other luminescent probes, and the vast range of temperatures that Ln^3+^ ions can be susceptible to, make Ln^3+^-doped materials great nanothermometer candidates [33].

The fluorescence intensity ratio (FIR) technique is the most frequently used method for optical temperature sensing. In single-center radiometric thermometers, the fluorescence intensity ratio (FIR) technique is defined using the emission intensity ratio I_2_/I_1_ from two thermally coupled electronic levels, with the requirement that they must be energy-close transitions, in the range from 200 to 2000 cm^−1^, where I_1_ and I_2_ are the integrated luminescence intensities of the lower (1) and higher (2) energy levels, respectively [35,36]. *FIR* depends on a Boltzmann energy transfer distribution between the (1) and (2) levels, and can be expressed with the following equation [34]:

(1) $$FIR=\frac{I_{2}}{I_{1}}=Aexp\left(-\frac{\Delta E}{K_{B\;}T}\right)$$

where *A* is a constant that depends on the degeneracies, populations, total spontaneous emission rates, and the frequencies of the two levels. Moreover, ∆*E* (cm^−1^) is the energy gap between the barycenter of the involved levels, *K_B_* (cm^−1^ per K) is the Boltzmann constant, and *T* (K) is the absolute temperature. The FIR technique has the following advantages: high spatial resolution, high thermal resolution, fast response time, and wide operating temperature range [34]. Additionally, FIR is independent of external disturbances, such as luminescence loss, fluctuations in the excitation source during the temperature measurement, and response curve of the detector [37].

Here, we study TiO_2_ nanoparticles doped with Nd^3+^ ions (Nd^3+^–TiO_2_), synthesized by the sol–gel method, reported as having high reproducibility and high purity and homogeneity of the products [18]. The Nd^3+^ doping is ideal for biological applications, as excitation and emission occur in biological windows (biological windows (BW) are reported from 650 to 950 nm, from 1000 to 1380 nm, and, the third, from 1500 to 1800 nm), avoiding cell auto-luminescence [30]. The quantity of the dopant Nd^3+^ was varied in low concentrations, from 0.5, 1 and 3 wt.%, in order to avoid luminesce quenching, as observed in high doping amounts, clustering of Nd at the nanoparticle surface, and possible increase in the toxicity of the Nd^3+^–TiO_2_ [30,38,39]. The morphology, phase, and chemical composition of the synthesized nanoparticles was characterized by XRD, SEM-EDXS, TEM, HRTEM, XPS and NEXAFS techniques. The photoluminescence of the nanoparticles was investigated using 350, 514 and 600 nm as excitation wavelengths. In order to observe the dependence on temperature of the luminescence intensity of the Nd^3+^–TiO_2_ nanoparticles, their luminescent spectra, in the range between 700 and 950 nm, were acquired after excitation at 600 nm at different temperatures (288–348 K), without using the correction response curve of the R928 photomultiplier, allowing the determination of the FIR values with accuracy. The relative sensitivity of the nanoparticles was obtained using the fluorescence intensity ratio between the Nd^3+^ following transitions: I821I880=F45/2→I49/2F43/2→I49/2 and I760I880=F47/2→I49/2F43/2→I49/2, as reported by Balabhadra et al. 2015 [40].

## 2. Experimental Section

### 2.1. Materials Synthesis

The synthesis of TiO_2_ nanoparticles doped with neodymium was carried out using a modified sol–gel method adapted from Sugimoto et al. [41]. The following four batches of nanoparticles were synthesized varying the neodymium content: 0.5, 1 and 3 wt.% and a control batch, undoped TiO_2_ (0% Nd). First, a solution of titanium isopropoxide (Ti(OiPr)_4_) was prepared by diluting 15.4 mL of (Ti(OiPr)_4_) with 250 mL of absolute ethanol (EtOH(abs)). Then, a solution of neodymium salt (Nd(NO_3_)_3_ • 6H_2_O) was prepared by dissolving a calculated amount of Nd(NO_3_)_3_ • 6H_2_O (61.1 mg (0.5 wt.%), 112.8 mg (1 wt.%) and 375.9 mg (3 wt.%)) in 40 mL of EtOH(abs). When the salt was fully dissolved 1.3 mL of triethanolamine was added. The prepared solution was added to the solution of Ti(OiPr)_4_. The mixture was stirred at room temperature for 5 min and then a solution of 40 mL of EtOH and 10 mL of deionized water was added dropwise. The mixture was stirred at room temperature for 3 h. In a next step, the reaction mixture was dried on a magnetic stirrer at 70 °C for 5 h, then at 100 °C for 12 h and in the oven at 250 °C for 12 h, followed by calcination at 550 °C for 10 h. The doped samples were labeled 0-Nd–TiO_2_ (undopped), 0.5-Nd–TiO_2_, 1-Nd–TiO_2_ and 3-Nd–TiO_2_ where the first number denotes a neodymium doping level. 

### 2.2. Materials Characterization: Morphology, Structural and Elemental

A D4 Endeavor, Bruker AXS diffractometer with Cu Kα radiation (λ = 1.5406 Å) and a Sol-X energy-dispersive detector was used to record XRD (X-ray diffraction) patterns, for phase composition determination. The diffractograms were acquired with collection time of 3 s in the 2θ angular range with a step size of 0.02° s^–1^.

A field emission scanning electron microscope (FE-SEM, Jeol 7600F) and a transmission electron microscope (TEM Jeol 2100, 200 keV) were used to determine the morphology of the nanoparticles. The samples were dispersed in water and supported on an Al sample holder for SEM analysis, a 5-nm-thick carbon layer was deposited on the sample to avoid build-up of charge during the SEM analysis. For the TEM analyses, the samples were dispersed ultrasonically in methanol and a drop of the dispersion was put onto a copper grid supporting a lacy carbon film (300 mesh).

Neodymium content was determined from EDX spectroscopy (energy-dispersive X-ray) combined with the FE-SEM. For the EDXS analysis (FE-SEM) the powder samples were pressed into pellets and placed on a carbon tape on an Al sample holder and coated with a thin carbon layer prior to the analyses.

The elemental composition of the surface of the nanoparticles was analyzed by X-ray photoelectron spectroscopy (XPS). From the analysis of the XPS spectra we also obtained the chemical states of titanium in the samples through the curve-fitting analysis of Ti*2p* XPS spectrum using the CASA XPS software. A VersaProbe PHI 5000 spectrometer from Physical Electronics, equipped with a monochromatic Al Kα X-ray source with energy resolution of 0.5 eV, the X-ray beam diameter 200 µm was used to record the XPS spectra. During the measurements, a dual-beam charge neutralization with an electron gun (1 eV) and an Ar ion gun (≤10 eV) were used for charge compensation of the sample surface. The C1s peak centered at 284.6 eV was used for binding energy reference.

The TXM (transmission X-ray microscope) end-station equipped with a 25 nm zone plate installed at the undulator beamline U41-XM at the electron storage ring BESSY II, Helmholtz-Zentrum Berlin (HZB) was used to record the NEXAFS spectra [42,43]. The calculated spectral resolution was *E*/Δ*E* = 20000. The photon flux variation with photon energy (hv) and acquisition time used for recording the different regions of the spectra were corrected using a spectrum recorded in bare regions near the nanoparticle. The axis2000 was used for data analysis (A.P.Hitchock, http://unicorn.mcmaster.ca/aXis2000.html) (accessed on 10 March 2021).

### 2.3. Optical Characterization

The near-infrared photoluminescence spectra were recorded in the range of 725 to 1000 nm and from 800 to 1450 nm using an argon laser (Coherent Innova 400, 514 nm) as the excitation source. The photoluminescent signals were dispersed by a Thermo Jarrel Ash monochromator (0.3 m, model 82497) coupled with a photomultiplier (Hamamatsu/R632) (for the range from 725 to 1000 nm) and an InGaAs sensor (for the range from 800 to 1450 nm) for detection. The Nd^3+^ luminescence decay time was monitored at 1096 nm resulting in the ^4^F_3/2_→^4^I_11/2_ transition, using the Thermo Jarrel Ash monochromator (0.3 m) and the InGaAs detector; the second harmonic of a Nd: YAG laser (Surelite/Continumm, 532 nm, 10 Hz, 5 ns) was used as an excitation source. A digital oscilloscope (Tektronix/TDS380) was used to record the decay time curves.

A SPEX Fluorolog spectrofluorometer (0.22 m, Spex/1680) (excitation source = Xe-lamp, detection = photomultiplier (Hamamatsu/R928)) was used to record the photoluminescence spectra in the visible region. Further details can be found in Borrero-González et al. 2020 [35]. The optical emission of the defect band in TiO_2_ in the range from 380 to 750 nm was recorded after excitation at 350 nm at room temperature. The photoluminescence spectra in the range from 700 to 950 nm were recorded at different temperatures after excitation at 600 nm. The temperature of the samples was varied in the range of 288–348 K using a Peltier cooling/heating homemade system equipped with a temperature controller (AUTONICS-TZN4S), with resolution of 0.10 K.

## 3. Results and Discussion

**Figure 1:**
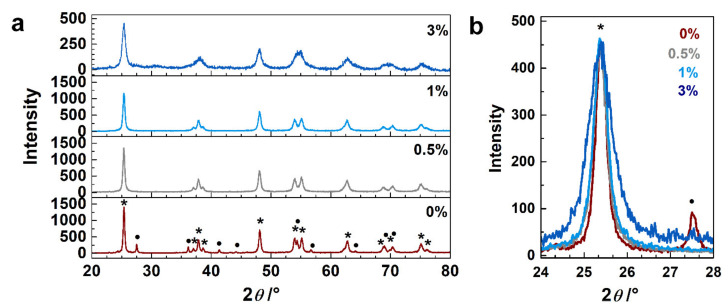
(**a**) Powder XRD patterns of undoped TiO_2_ sample and samples doped with 0.5, 1 and 3 wt.% of Nd^3+^. (**b**) Enlarged (101) peak region; for all samples the intensity of the (101) peak was normalized to the peak of the 3-Nd–TiO_2_. Peaks marked with * stand for anatase while • for rutile.

The synthesized samples were characterized in order to determine their crystallographic phase composition, morphology, doping level, and oxidized states of the neodymium and titanium atoms. Figure 1a compares the diffractograms of the undoped and Nd^3+^-doped (0.5, 1 and 3 wt.%) TiO_2_ samples. The XRD pattern of the undoped TiO_2_ exhibits diffraction peaks of anatase and rutile TiO_2_ (matching ICCD cards no. 86-1157 and 87-0710, respectively); the estimated amount of rutile in the sample is about 20%, (calculated using Spurr’s formula (Spurr, 1957)). The XRD patterns of the neodymium-doped TiO_2_ samples match to anatase TiO_2_ (ICCD card no. 86-1157). Comparing the diffractograms of the doped samples, we can observe that (i) the intensity of the (101) peak decreases with an increasing amount of neodymium (Figure 1a), and (ii) the peak width becomes wider with an increasing amount of dopant (Figure 1b). These results indicate that the presence of Nd^3+^ (i) suppressed the transformation of the anatase phase to rutile, at processing conditions as reported for Gd–La-doping of TiO_2_ (Wang 2015), and/or (ii) it caused disorder in the TiO_2_ lattice that can be related to the difference in the ion radii of Ti^4+^ and Nd^3+^ (0.64 Å and 0.98 Å, respectively). The doping with Nd^3+^ was reported to create oxygen vacancies in TiO_2_, to maintain the neutrality after the introduction of Nd^3+^ (for each two Nd^3+^ introduced, one O^2-^ is released) [44]. Disorder can be caused by occupation of interstitial sites; however, the interstitial sites in TiO_2_ are smaller than the ion radii of Nd^3+^, which is a strong reason that may impede the occupation of Nd^3+^ in interstitial position. Moreover, this would also involve the accommodation of O^2-^ ions in the vicinity of the interstitial Nd^3+^, to compensate the charges, and a considerable number of Ti^4+^ vacancies would be created [44]. Therefore, besides the creation of defects, there is the possibility that part of the Nd^3+^ remains on the surface of TiO_2_ [22]; however, no peaks that were associated with the formation of neodymium oxide particles were observed for the low Nd doping amounts used. The (101) peak position in the doped samples is slightly shifted toward lower angles (2θ~25.36°) compared to the undoped sample (2θ~25.39°), indicating a slight increase in the unit cell as a consequence of Nd^3+^ doping. Table S1 compares the doping with the variation in the band energy gap; it can be seen that at low concentrations, the optical gap did not vary noticeably.

**Figure 2:**
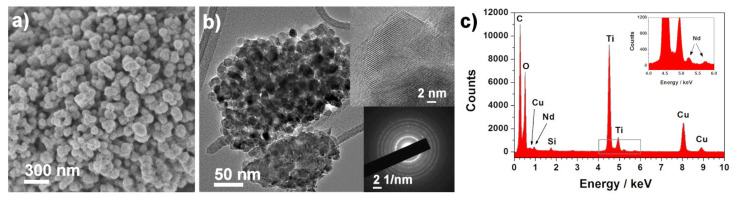
(**a**) SEM and (**b**) TEM images with corresponding SEAD pattern (bottom inset), and (**c**) EDX spectrum of the 3-Nd–TiO_2_ sample. EDX spectrum (**c**) was taken over an area presented in Image (**b**). Upper inset in Image (**b**) shows a crystalline particle, the interlayer distance is 0.37 nm.

Morphological characterization was carried out for all the synthesized samples, but, for simplification, only the 3 wt.% Nd^3+^–TiO_2_ results are shown (Figure 2); SEM and TEM investigation of 3-Nd–TiO_2_ revealed that the sample consists of round-like structures, with diameters between 150 nm and 300 nm (Figure 2a), which are composed of aggregated nanoparticles with diameters around ~15–20 nm (Figure 2b). The powder sample is polycrystalline, as revealed by the selected electron area diffraction (SEAD) pattern (Figure 2b, bottom inset). The EDX spectrum (Figure 2c), taken over the same area as presented in Figure 2b, shows the presence of neodymium. The measured d-spacing of the lattice (101) planes is 0.37 nm, and is slightly increased when compared to 0.35 nm for anatase (ICDD 78-2486 pattern). The neodymium content, determined with EDXS analysis using SEM, was 0.6 wt.% ± 0.3 (0.5-Nd–TiO_2_), 0.9 wt.% ± 0.2, (1-Nd–TiO_2_) and 2.9 wt% ± 0.4 (3-Nd–TiO_2_).

**Figure 3:**
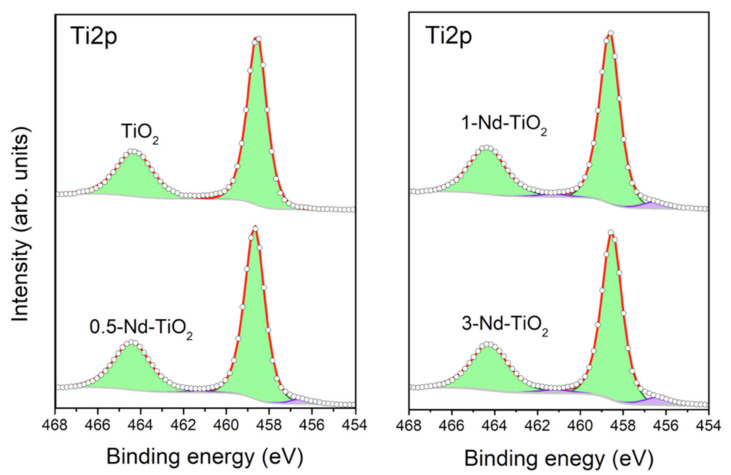
Curve-fitting XPS analysis of high-resolution Ti*2p* spectrum from 0, 0.5, 1 and 3 wt.% Nd-doped TiO_2_. The green doublet is assigned to titanium with oxidation state Ti^4+^ and the purple doublet to Ti^3+^.

X-ray photoelectron spectroscopy was used to determine the surface chemical composition of the nanoparticles, and the oxidation state of the titanium in the samples. The survey spectra of the samples show the presence of titanium, oxygen, carbon, and neodymium (Supplementary Materials Figure S1). The presence of carbon is related to contamination due to air exposure. The curve-fitting analysis of the high-resolution Ti*2p* spectra of undoped, 0.5, 1 and 3 wt.% Nd–TiO_2,_ shown in Figure 3, provides information on the chemical states of the titanium in the samples. Because of the *j–j* coupling or spin–orbit splitting of the Ti*2p* level, each state of oxidation of titanium has two components in the spectrum, named as the doublet, one corresponding to Ti*2p_3/2_* and another to Ti*2p_1/2_*. To reproduce the Ti*2p* spectra of the three Nd–TiO_2_ samples, two doublets were used. The doublet with binding energies at 458.5 and 464.2 eV (green doublet) is assigned to the titanium with an oxidation state Ti^4+^ [45]. The doublet with binding energies at 456.3 and 461.1 eV (purple doublet) was reported to be associated with Ti^3+^. The relative contribution of this component to the Ti*2p* spectrum increases for increasing Nd^3+^ content, from 3.1% of the total amount of titanium in the 0.5-Nd–TiO_2_ sample, followed by 5.2% in the sample 1-Nd–TiO_2_, to a relative concentration of 6.0% in the sample 3-Nd–TiO_2_. It has been reported that even in TiO_2_ with high crystallinity, a minor amount of Ti^3+^, due to oxygen vacancies, is present [46,47,48]. Therefore, in the samples, the presence of reduced Ti^3+^ can be associated with oxygen defects, which increase with an increasing amount of Nd^3+^, indicating the introduction of oxygen defects in the TiO_2_ lattice when Nd^3+^ is added as the dopant, which is in agreement with the XRD results, and as reported in [49]. As expected, metallic titanium (Ti^0^) was not founded in the samples.

Due to the small amount of Nd atoms in the TiO_2_ matrix, the evaluation of the Nd oxidation state from the Nd XPS spectra is not straightforward. Alternatively, as X-ray absorption of rare earths in the region of the 3d level have high-absorption cross sections, and the energy position of the X-ray absorption edges are very sensitive to changes in the valence state, NEXAFS-TXM allows the evaluation of the oxidation state of rare earths in doping quantities [50,51]. Figure S2 shows the Nd M_4,5_ NEXAFS-TXM spectrum for the 0.5-Nd–TiO_2_ sample, the Nd M_4_-edge is at 1000.3 eV and the Nd M_5_-edge at 978.5 eV, and these energy positions were reported to be characteristic of the Nd^3+^ [50].

**Figure 4:**
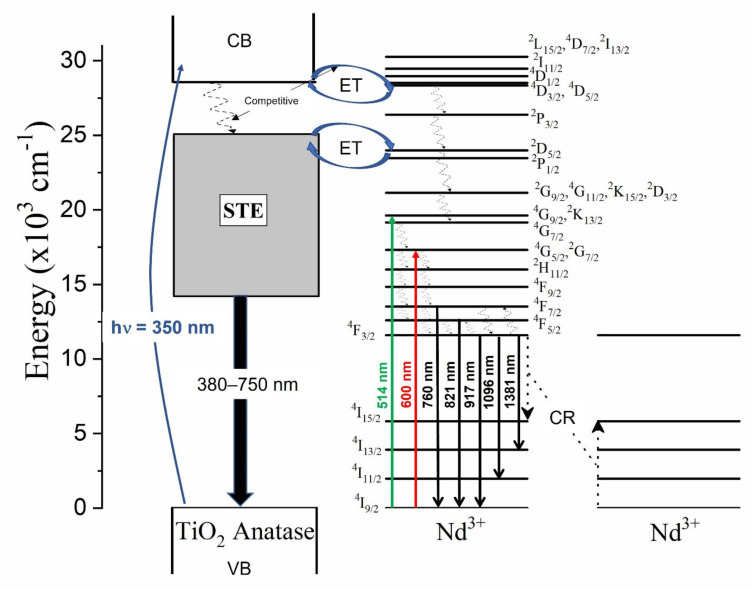
Energy level diagram of Nd^3+^–TiO_2_ under excitation at 350, 514 and 600 nm presenting the radiative, energy transfer, non-radiative and cross-relaxation process.

The luminescence properties of the undoped and the Nd^3+^–TiO_2_ nanoparticles were investigated, Figure 4 shows the schema of the excitation, radiative emission, energy transfer, non-radiative emission, and cross-relaxation (CR) processes affecting the photoluminescence of this nanomaterial. These processes are described as follows: excitation at 350 nm (~3.5 eV), above the energy band gap of TiO_2_ (~3.2 eV), promotes the transition of electrons from the valence band (VB) to the conduction band (CB) in TiO_2_. Then follows energy transfer (ET) from CB to the excited states of the Nd^3+^ ions, due to resonance in the energy levels. In parallel, non-radiative relaxation occurs, from CB to defect bands within the band gap of TiO_2_. Both the mechanisms are competitive, with a strong dependence on temperature [14]. Radiative relaxation from the defect bands to the valence band may occur, generating a broad emission band from 380 to 750 nm (see Figure S2). As can be observed, the emission that is associated with the defects has two components for all the samples, with a maximum intensity at 512 nm for the 0.5-Nd–TiO_2_ sample, while for the 1-Nd–TiO_2_ and 3-Nd–TiO_2_ samples, there is a spectral shift in the maxima to higher energy centered at 430 nm; these results are in agreement with the literature [52,53]. The intensity of the defect emission band increases for increasing Nd^3+^ concentration. As shown by XRD (Figure 1) and XPS (Figure 3), the doping with Nd^3+^ creates oxygen vacancies in TiO_2_, which is, therefore, in agreement with the literature, and the long-wavelength defect band emission can be attributed self-trapped excitons (STE) associated with oxygen vacancies [11,14,51,53]. The STE emission intensity of TiO_2_ increases severally with the increase in Nd^3+^ concentration, due to the increasing number of oxygen vacancies, and also the increasing concentration of Ti^3+^ in the nanoparticles, which is in agreement with the XPS analysis of the Ti*2p* core level. 

In TiO_2_ doped with Nd^3+^ ions, the non-radiative relaxation occurs via multiphonon, generating near-infrared emissions that are associated with the ^4^F_7/2_→^4^I_9/2_ (760 nm), ^4^F_5/2_→^4^I_9/2_ (821 nm), ^4^F_3/2_→^4^I_9/2_ (917 nm), ^4^F_3/2_→^4^I_11/2_ (1096 nm), and ^4^F_3/2_→^4^I_13/2_ (1381 nm) transitions. The decrease in the intensity of these transitions with the increasing Nd^3+^ concentration is associated with a well-known cross-relaxation (CR) process between adjacent Nd^3+^ ions in the host matrix (Figure 4). CR processes are dipolar transitions between Nd^3+^ ions, in the following way: the ^4^F_3/2_→^4^I_15/2_ transition of one Nd^3+^ ion resonantly transfers energy to a neighboring Nd^3+^ ion, causing the ^4^I_9/2_→^4^I_15/2_ excitation, and the electrons in the excited state of ^4^I_15/2_ of Nd^3+^ ions undergo a non-radiative relaxation to the ^4^I_9/2_ ground state of Nd^3+^. The normalized near-infrared photoluminescence spectra, in the range from 800 to 1450 nm of the *x*-Nd–TiO_2_, for *x* = 0.5, 1.0 and 3.0 wt.% under 514 nm excitation, are shown in Figure S3. The intensities of the spectra were corrected to the nominal Nd^3+^ concentration. The emission bands that are centered at 917, 1096 and 1381 nm can be respectively assigned to the ^4^F_3/2_→^4^I_9/2_, ^4^F_3/2_→^4^I_11/2_, ^4^F_3/2_→^4^I_13/2_ electronic transitions of the Nd^3+^ ion. The identified transitions correspond well with the reported data of Nd^3+^ in the anatase matrix [53,54]. The photoluminescence decay curves of the *x*-Nd–TiO_2_ (*x* = 0.5, 1.0 and 3.0 wt.%), detected at 1096 nm, due to ^4^F_3/2_→^4^I_11/2_ transition, were recorded after pulsed excitation at 532 nm (not shown here). All the transients exhibit non-exponential behavior; the average decay times decreased from τ=45 μs (0.5-Nd–TiO_2_) to τ=32 μs (3.0-Nd–TiO_2_), and these characteristic decay times confirm the presence of the CR process in the prepared samples, as depicted in Figure 4 [54,55].

**Figure 5:**
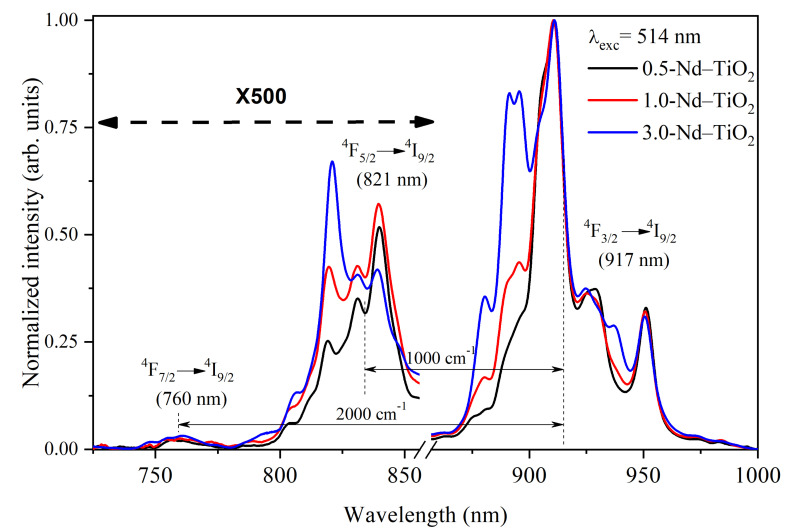
Normalized near-infrared photoluminescence spectra of *x*-Nd–TiO_2_ (*x* = 0.5, 1.0 and 3.0 wt.%) nanoparticles, excitation at 514 nm. The intensity of the ^4^F_7/2_→^4^I_9/2_ and ^4^F_5/2_→^4^I_9/2_ transitions were multiplied by a factor of 500 for better comparison.

The near-infrared photoluminescence spectra of the *x*-Nd–TiO_2_ nanoparticles, obtained using a 514 nm wavelength of excitation, and collected in the range from 725 to 1000 nm using the photomultiplier R632, are presented in Figure 5. For all the samples, three different emissions were observed, centered at 760, 821 and 917 nm, and these emissions are related to the intra-*4f* transitions of ^4^F_7/2_→^4^I_9/2_, ^4^F_5/2_→^4^I_9/2_ and ^4^F_3/2_→^4^I_9/2_ of Nd^3+^ ions, respectively. All these spectra were normalized at 911 nm for better visualization. 

The intensity of the ^4^F_7/2_→^4^I_9/2_, ^4^F_5/2_→^4^I_9/2_ transitions at 760 nm and 821 nm, respectively, are very low, due to the strong multiphonon relaxation from the ^4^F_7/2_ and ^4^F_5/2_ levels to the ^4^F_3/2_ level. Therefore, they were multiplied by a factor of 500 for better comparison. As can be observed, the intensity at 821 nm is about 500-fold lower than the intensity at 911 nm, and the intensity at 760 nm is even lower. Due to the large difference in the intensities of these transitions, the spectra shown in Figure 5 were taken at a high integration time, which prevents their use as a nanothermometer when a fast response time is needed. In addition, the large intensity difference in these transitions introduces inaccuracy into the determination of the FIR values. Therefore, to overcome this drawback, the emission spectra in the range between 700 and 950 nm, recorded at different temperatures in the range between 288 and 348 K, were acquired, without using the correction response curve of the R928 photomultiplier [40]. This photomultiplier has a large gain difference in the range between 800 and 900 nm (with high gain at 800 nm and low gain at 900 nm) Please replace by (https://www.hamamatsu.com/resources/pdf/etd/R928_R928P_R955_R955P_TPMS1091E.pdf (accessed on 10 March 2021) please check spectral responsed of R928). In this regard, the transitions that have low intensity (^4^F_7/2_→^4^I_9/2_, ^4^F_5/2_→^4^I_9/2_) are observed with high gain, and the transition of high intensity (^4^F_3/2_→^4^I_9/2_) is observed with low gain, i.e., the three transitions are observed in the same detection scale as shown below. This acquisition strategy of the photoluminescence spectra does not affect the calculated FIR values, since FIR is defined through a ratio of intensities.

**Figure 6:**
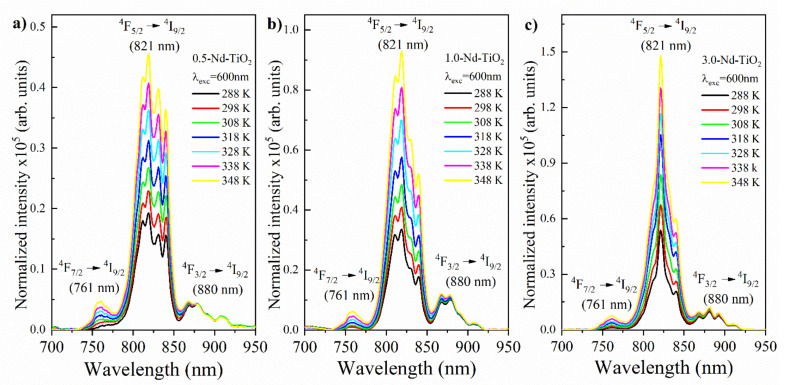
Temperature evolution of the emission spectra of (**a**) 0.5-Nd–TiO_2_, (**b**) 1-Nd–TiO_2_ and (**c**) 3-Nd–TiO_2_ after excitation at 600 nm. The ^4^F_3/2_→^4^I_9/2_ transition intensity at 880 nm was normalized for all samples. The spectra were not corrected for the detector response curve. All spectra were acquired with integration time equal to 1 s.

In order to evaluate the synthetized nanoparticles as temperature sensors, the luminescence spectrum for all three of the Nd^3+^–TiO_2_ samples were obtained using an excitation of 600 nm. The excitation power was 200 μW, to avoid uncontrolled extra heating of the nanothermometer. The spectra were taken at different controlled temperatures, in the range of 288–348 K (Figure 6). The luminescence intensity of the ^4^F_7/2_→^4^I_9/2_ and ^4^F_5/2_→^4^I_9/2_ transitions increase as the temperature increases. Conversely, the luminescence intensity of the ^4^F_3/2_→^4^I_9/2_ transition decreases as the temperature increases. For better visualization, the spectra were normalized to the ^4^F_3/2_ →^4^I_9/2_ transition intensity at 880 nm (this transition is centered at 917 nm; however, due to the low gain of the photomultiplier above 900 nm, this region of the emission spectrum is not clearly observed). The results have a reproducibility higher than 99%, as expected for a luminescent thermometer based on inorganic host doped with lanthanide ions, due to their stability in the studied temperature range.

**Figure 7:**
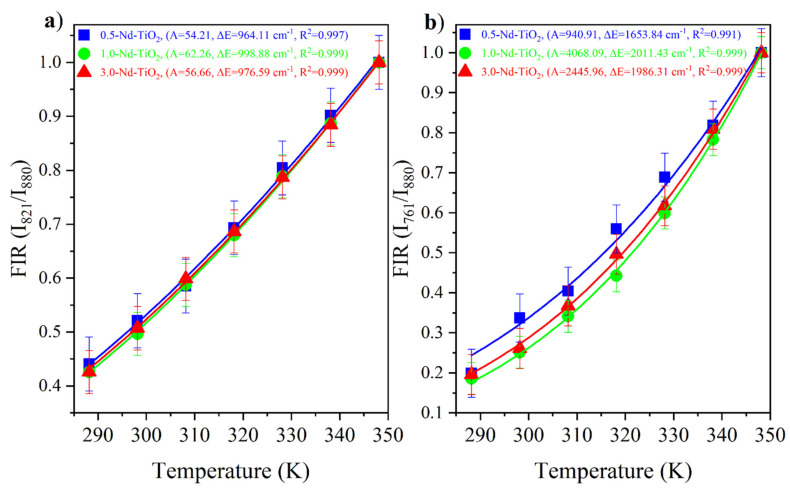
FIR values of *x*-Nd–TiO_2_ (*x* = 0.5, 1.0 and 3.0 wt.%) nanoparticles for the I821I880=F45/2→I49/2F43/2→I49/2 ratio (**a**) and the I760I880=F47/2→I49/2F43/2→I49/2 ratio (**b**). The FIR values are represented as symbols and FIR fitting is represented as continuous lines.

The Nd^3+^:^4^F_3/2, 5/2, 7/2_ are thermally coupled levels, the ratio between the I821I880=F45/2→I49/2F43/2→I49/2 and I760I880=F47/2→I49/2F43/2→I49/2 were obtained from the experimental data and fitted with Equation (1). The $\frac{I_{821}}{I_{880}}$ and $\frac{I_{760}}{I_{880}}$ FIR of the *x*-Nd–TiO_2_ (*x* = 0.5, 1.0 and 3.0 wt.%) nanoparticles are showed in Figure 7a and Figure 7b, respectively. The fitted values of A, ΔE, and R^2^ values are also shown in the same figures.

The FIR increases as the temperature increases for all the samples. The ∆E values from the fitting, ∆E = 1000 cm^−1^ for the energy separation between the ^4^F_5/2_ and ^4^F_3/2_ levels, and ∆*E*~2000 cm^−1^ for the energy separation between the ^4^F_7/2_ and ^4^F_3/2_ levels, are in total agreement with the corresponding values from the barycenter concerning the same levels as shown in Figure 5. In addition, good-quality fittings were obtained for all the samples. 

The figure of merit for the comparison of the performance of different thermometers is the relative thermal sensitivity (*S_r_*), calculated using Equation (2) [34].

(2) $$\;S_{r}=\;\frac{1}{FIR}\left|\frac{dFIR}{dT}\right|\left(\%K^{-1}\right)=\frac{\Delta E}{k_{B}T^{2}}\left(\%K^{-1}\right)$$

**Figure 8:**
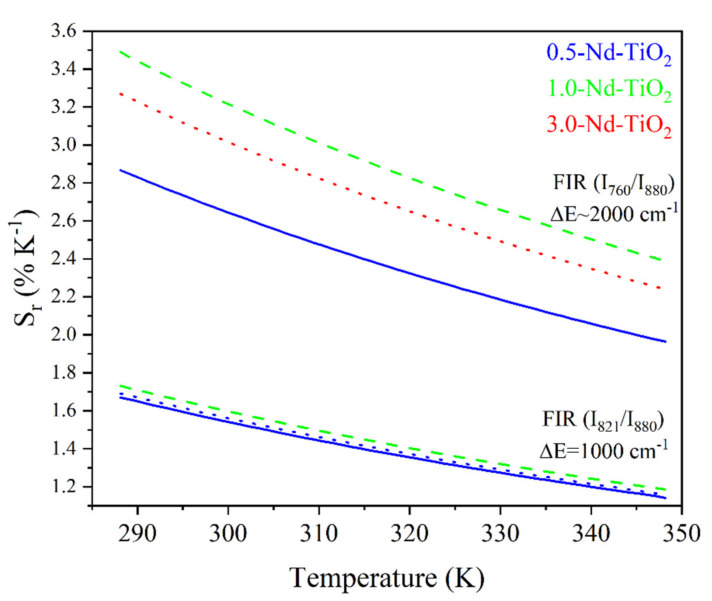
Relative thermal sensitivity values for *x*-Nd–TiO_2_ (*x* = 0.5, 1.0 and 3.0 wt.%) nanoparticles. The $\frac{I_{821}}{I_{880}}$ FIR and $\frac{I_{760}}{I_{880}}$ FIR are represented as solid blue, for *x* = 0.5, dash green, for *x* = 1.0 and dot red lines, for *x* = 3.0 wt.%.

The *S_r_* for the $\frac{I_{821}}{I_{880}}$ and $\frac{I_{760}}{I_{880}}$ *FIR* of the *x*-Nd–TiO_2_ (*x* = 0.5, 1.0 and 3.0 wt.%) nanoparticles are showed in Figure 8.

As can be observed, the relative thermal sensitivity decreases as the temperature increases; this phenomenon was observed for all the samples, as was expected from Equation (2) for thermally coupled levels. For the 0.5-Nd–TiO_2_ sample, the relative sensitivity shows the maximum value of 1.64 %K^−1^ at 288 K and the minimum value of 1.16 %K^−1^ at 348 K, when the I_821_/I_880_ FIR was used. On the other hand, a maximum value of 2.67 %K^−1^ at 288 K and a minimum of 2.07 %K^−1^ at 348 K were calculated when the I_760_/I_880_ FIR was used. For the case of the 1-Nd–TiO_2_ sample, the relative sensitivity shows the maximum value of 1.73 %K^−1^ at 288 K and a minimum value of 1.19 %K^−1^ at 348 K, when the I_821_/I_880_ FIR was used. Conversely, a maximum value of 3.43 %K^−1^ at 288 K and a minimum of 2.38 %K^−1^ at 348 K were calculated when the I_760_/I_880_ FIR was used. Alternatively, the relative sensitivity of the 3.0-Nd–TiO_2_ shows a maximum value of 1.67 %K^−1^ at 288 K and a minimum value of 1.17 %K^−1^ at 348 K for the I_821_/I_880_ FIR. Secondly, a maximum value of 3.04 %K^−1^ at 288 K and a minimum of 2.32 %K^−1^ at 348 K were calculated for the I_760_/I_880_ FIR. The 3.0-Nd–TiO_2_ sample had the higher intensity and the best signal-to-noise ratio; however, the *S_r_* values were lower, which may be associated with the cross-relaxation process, which is more effective at high Nd^3+^ concentrations, as was shown in Figure S3. The 1.0-Nd–TiO_2_ sample presented the best *S_r_* values. However, the 0.5-Nd–TiO_2_ sample did not follow the expected sequence of this set of samples, which could be associated with the low R^2^ values as shown in Figure 7.

The calculated *S_r_* values between 3.43 and 1.16 %K^−1^, in the temperature range from 288 to 348 K, are higher than those reported in the literature. For example, in the work of Rocha et al., *S_r_* decreases from 0.083 to 0.070 %K^−1^ when the temperature increases from 303 to 330 K, in Nd^3+^:LaF_3_ nanoparticles [56]. These low *S_r_* values can be associated with the use of emissions from the Stark energy sublevels (R_1_ and R_2_) of ^4^F_3/2_ (see Figure 5b in [55], where R_1_ = 11577 cm^−1^ and R_2_ = 11630 cm^−1^ with an energy difference ΔE=53 cm^−1^, which is considerably lower than the difference in energy of the levels used in this work, ∆E~1000 cm^−1^ or 2000 cm^−1^). More recently, Silva et al. reported a decrease in *S_r_* values, from 0.83 to 0.30 %K^−1^, in the temperature range from 300 to 353 K in Nd^3+^:TiO_2_ highly doped nanocrystals [30]. Therefore, to the best of our knowledge, the *S_r_* values that have been reported in this work are the highest in a Nd^3+^-doped TiO_2_ nanothermometer, so far.

**Figure 9:**
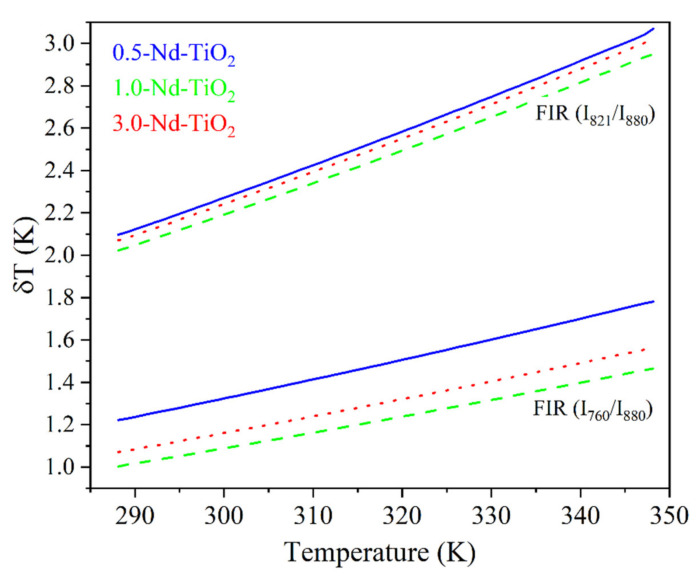
Thermal resolution calculated using Equation (3) of *x*-Nd–TiO_2_ (*x* = 0.5, 1.0 and 3.0 wt.%) nanoparticles.

A relevant parameter for the performance of the thermometers, and related to *S_r_*, is the temperature uncertainty, $\delta T\left(T\right)$ (also called thermal resolution), given by Bednarkiewicz et al. [57].

(3) $$\delta T\left(T\right)=\frac{1}{S_{r}}\left|\frac{\delta \left(FIR\right)}{FIR}\right|$$

where, $\frac{\delta \left(FIR\right)}{FIR}$ ~3.5% is the relative error in the *FIR* determination, which was estimated from our acquisition setup, for an integration time of 1 s. Figure 9 shows δT for the *x*-Nd–TiO_2_ nanoparticles. 

Further, δT monotonically increases for all the samples in the temperature range from 288 to 348 K, as expected from Equation (3). The better thermal resolution is obtained when the I_821_/I_880_ FIR was used; however, the δT values can be improved by increasing the integration time of the emission spectra.

## 4. Conclusions

The synthesis of TiO_2_ nanoparticles doped with 0.5, 1 and 3 wt.% of Nd^3+^ was successfully achieved via the sol–gel method. The crystal phase was anatase for the three doped samples, unlike undoped TiO_2_, which was founded to have a mixture between the rutile and anatase phases. The oxidation state of titanium is mostly Ti^4+^, with a small contribution of Ti^3+^, which has a relative concentration that increases as the Nd concentration increases in the samples; only Nd^3+^ was detected in all three of the *x*-Nd–TiO_2_ samples. Defects, such as oxygen vacancies and Ti^3+^ created after the Nd^3+^ incorporation into the TiO_2_ matrix, generated electronic states (defect states), and the emission originating in these states explains the emission of TiO_2_ in the visible region. The cross-relaxation effect between the Nd^3+^ neighboring ions was evidenced from the near-infrared emissions, and this effect became more efficient with the increasing Nd^3+^ concentration. The Nd^3+^:^4^F_3/2_→^4^I_9/2_ (880 nm), ^4^F_5/2_→^4^I_9/2_ (821 nm), and ^4^F_7/2_→^4^I_9/2_ (760 nm) transitions are within the first biological window of tissues, and showed a strong dependence on temperature in the physiological temperature range, indicating the high potential of this nanomaterial in biological applications. The Nd^3+^:^4^F_3/2_→^4^I_9/2_ (880 nm), ^4^F_5/2_→^4^I_9/2_ (821 nm), and ^4^F_7/2_→^4^I_9/2_ (760 nm) transitions are not commonly used in the FIR technique, due to the strong multiphonon relaxation from the ^4^F_7/2_ and ^4^F_5/2_ to the ^4^F_3/2_ level, leading to a large difference in the intensities from these transitions, and this could give inaccurate results. However, the use of a photomultiplier tube R928 without the response curve of wavelength dependence permitted the observation of comparable intensities from these transitions, which permits the construction of more accurate nanothermometers, with fast response times. The calculated relative thermal sensitivities are higher than those reported in the literature. The reported performance of the nanothermometers shows a strong dependence on the Nd^3+^ concentration, with the 1.0-Nd–TiO_2_ nanothermometer being the best, achieving a relative thermal sensitivity equal to 3.43 %K^−1^ and a thermal resolution equal to 1.02 K at 288 K, when the I_821_/I_880_ FIR was used.

## Data Availability

Raw data are available from authors upon request.

## Supplementary Materials

The following are available online at https://www.mdpi.com/article/10.3390/s21165306/s1, Figure S1: XPS survey spectrum recorded on 3-Nd-TiO_2_ sample, Figure S2: M-edges spectrum recorded on the 0.5-Nd-TiO_2_ sample. The horizontal axe gives the excitation photon energy in eV. The open circles are the experimental data and the red line a guide to eye, Figure S3: Luminescence spectra of *x*-Nd-TiO_2_ (*x* = 0.5, 1.0 and 3.0 wt%) nanoparticles, excitation at 350 nm and temperature of 298 K, Figure S4: Normalized infrared photoluminescence spectra of *x*-Nd-TiO_2_ (*x* = 0.5, 1.0 and 3.0 wt%) under 514 nm excitation. The spectra were corrected to the Nd^3+^ concentration. The average decay times (*x*) of each sample are shown in the legend, Table S1: Summarized data for phase composition, neodymium content and band gap for undoped and Nd^3+^-doped TiO_2_ samples.

## References

[1] Daghrir R., Drogui P., Robert D. (2013). Modified TiO_2_ for environmental photocatalytic applications: A review. Ind. Eng. Chem. Res..

[2] Khaki M.R.D., Shafeeyan M.S., Raman A.A.A., Daud W.M.A.W. (2017). Application of doped photocatalysts for organic pollutant degradation—A review. J. Environ. Manag..

[3] Grätzel M. (2000). Perspectives for dye-sensitized nanocrystalline solar cells. Prog. Photovolt. Res. Appl..

[4] Wojcieszak D., Mazur M., Kurnatowska M., Kaczmarek D., Domaradzki J., Kępiński L., Chojnacki K. (2014). Influence of Nd-doping on photocatalytic properties of TiO_2_ nanoparticles and thin film coatings. Int. J. Photoenergy.

[5] Domaradzki J., Mazur M., Sieradzka K., Wojcieszak D., Adamiak B. (2013). Photocatalytic properties of Ti-V oxides thin films. Opt. Appl..

[6] Rodríguez E.M., Rey A., Mena E., Beltrán F.J. (2019). Application of solar photocatalytic ozonation in water treatment using supported TiO_2_. Appl. Catal. B Environ..

[7] Tran V.A., Truong T.T., Phan T.A.P., Nguyen T.N., van Huynh T., Agresti A., Pescetelli S., Le T.K., di Carlo A., Lund T. (2017). Application of nitrogen-doped TiO_2_ nanotubes in dye-sensitized solar cells. Appl. Surf. Sci..

[8] Tugaoen H.O., Garcia-Segura S., Hristovski K., Westerhoff P. (2018). Compact light-emitting diode optical fiber immobilized TiO_2_ reactor for photocatalytic water treatment. Sci. Total Environ..

[9] Carbone R., Marangi I., Zanardi A., Giorgetti L., Chierici E., Berlanda G., Podestà A., Fiorentini F., Bongiorno G., Piseri P. (2006). Biocompatibility of cluster assembled nanostructured TiO_2_ with primary and cancer cells. Biomaterials.

[10] Wang Y., Wen C., Hodgson P., Li Y. (2013). Biocompatibility of TiO_2_ nanotubes with different topographies. J. Biomed. Mater. Res. Part A.

[11] Abazović N., Comor M., Dramicanin M., Jovanovic D., Ahrenkiel S.P., Nedeljkovic J. (2006). Photoluminescence of anatase and rutile TiO_2_ particles. J. Phys. Chem. B.

[12] Mazierski P., Mikołajczyk A., Bajorowicz B., Malankowska A., Zaleska-Medynska A., Nadolna J. (2018). The role of lanthanides in TiO_2_-based photocatalysis: A review. Appl. Catal. B Environ..

[13] Zaleska-Medynska A. (2008). Doped-TiO_2_: A review. Recent Pat. Eng..

[14] Le Boulbar E., Millon E., Ntsoenzok E., Hakim B., Seiler W., Boulmer-Leborgne C., Perrière J. (2012). UV to NIR photon conversion in Nd-doped rutile and anatase titanium dioxide films for silicon solar cell application. Opt. Mater..

[15] Choudhury B., Choudhury A. (2012). Dopant induced changes in structural and optical properties of Cr^3+^ doped TiO_2_ nanoparticles. Mater. Chem. Phys..

[16] Kaleji B.K., Sarraf-Mamoory R., Fujishima A. (2012). Influence of Nb dopant on the structural and optical properties of nanocrystalline TiO_2_ thin films. Mater. Chem. Phys..

[17] Singh J., Sharma S., Sharma S., Singh R.C. (2019). Effect of tungsten doping on structural and optical properties of rutile TiO_2_ and band gap narrowing. Optik.

[18] Garskaite E., Flø A.S., van Helvoort A.T.J., Kareiva A., Olsen E. (2013). Investigations of near IR photoluminescence properties in TiO_2_: Nd, Yb materials using hyperspectral imaging methods. J. Lumin..

[19] Bünzli J.-C.G., Eliseeva S., Hänninen P., Härmä H. (2011). Basics of lanthanide photophysics. Lanthanide Luminescence.

[20] Liu F., Ma E., Chen D., Yu Y., Wang Y. (2006). Tunable red-green upconversion luminescence in novel transparent glass ceramics containing Er: NaYF_4_ nanocrystals. J. Phys. Chem. B.

[21] Quach A., Escax V., Nicole L., Goldner P., Guillot-Noël O., Aschehoug P., Hesemann P., Moreau J., Gourier D., Sanchez C. (2007). Rare earth doped mesoporous hybrid thin films with tunable optical responses. J. Mater. Chem..

[22] Wojcieszak D., Mazur M., Kaczmarek D., Morgiel J., Zatryb G., Domaradzki J., Misiewicz J. (2015). Influence of Nd dopant amount on microstructure and photoluminescence of TiO_2_: Nd thin films. Opt. Mater..

[23] Pandiyan R., Bartali R., Micheli V., Gottardi G., Luciu I., Ristic D., Alombert-Goget G., Ferrari M., Laidani N. (2011). Influence of Nd^3+^ doping on the structural and near-IR photoluminescence properties of nanostructured TiO_2_ films. Energy Procedia.

[24] Jia C., Xie E., Peng A., Jiang R., Ye F., Lin H., Xu T. (2006). Photoluminescence and energy transfer of terbium doped titania film. Thin Solid Film..

[25] Malakhovskii A., Gnatchenko S., Kachur I., Piryatinskaya V., Temerov V. (2016). Peculiarities of magnetic properties of Nd^3+^ ions in the Nd_0.5_Gd_0.5_Fe_3_(BO_3_)_4_ crystal in the optically excited states ^4^(F_7/2_ + S_3/2_) and (^4^G_9/2_ + ^2^K_13/2_ + ^4^G_7/2_). J. Alloy. Compd..

[26] Páez-Hernández D. (2016). Effect of the crystal environment on the optical and magnetic properties of Nd^3+^ and U^3+^ ions. Polyhedron.

[27] Vijayalakshmi L., Kumar K.N., Kumar G.B., Hwang P. (2017). Structural, dielectric and photoluminescence properties of Nd^3+^ doped Li_2_O-LiF-B_2_O_3_-ZnO multifunctional optical glasses for solid state laser applications. J. Non Cryst. Solids.

[28] Li W., Wang Y., Lin H., Shah S.I., Huang C.P., Doren D.J., Rykov S.A., Chen J.G., Barteau M.A. (2003). Band gap tailoring of Nd^3+^-doped TiO_2_ nanoparticles. Appl. Phys. Lett..

[29] Hassan M.S., Amna T., Yang O.-B., Kim H.-C., Khil M.-S. (2012). TiO_2_ nanofibers doped with rare earth elements and their photocatalytic activity. Ceram. Int..

[30] Silva W., Silva A., Rocha U., Dantas N., Jacinto C. (2021). Nd^3+^ doped TiO_2_ nanocrystals as self-referenced optical nanothermometer operating within the biological windows. Sens. Actuators A Phys..

[31] Jaque D., Vetrone F. (2012). Luminescence nanothermometry. Nanoscale.

[32] Dramićanin M.D. (2020). Trends in luminescence thermometry. J. Appl. Phys..

[33] Brites C., Lima P., Silva N., Millán A., Amaral V., Palacio F., Carlos L. (2012). Thermometry at the nanoscale. Nanoscale.

[34] Brites C., Balabhadra S., Carlos L.D. (2018). Lanthanide-based thermometers: At the cutting-edge of luminescence thermometry. Adv. Opt. Mater..

[35] Borrero-González L., Acosta S., Bittencourt C., Garvas M., Umek P., Nunes L.A.O. (2020). Eu^3+^-doped titanium oxide nanoparticles for optical thermometry in the first biological window. Opt. Mater..

[36] De Sá G., Malta O., de Mello Donegá C., Simas A., Longo R., Santa-Cruz P., da Silva E. (2000). Spectroscopic properties and design of highly luminescent lanthanide coordination complexes. Coord. Chem. Rev..

[37] Wade S., Collins S.F., Baxter G. (2003). Fluorescence intensity ratio technique for optical fiber point temperature sensing. J. Appl. Phys..

[38] Sontakke A., Biswas K., Mandal A.K., Annapurna K. (2010). Concentration quenched luminescence and energy transfer analysis of Nd^3+^ ion doped Ba-Al-metaphosphate laser glasses. Appl. Phys. A.

[39] Rim K.T., Koo K.H., Park J.S. (2013). Toxicological evaluations of rare earths and their health impacts to workers: A literature review. Saf. Health Work.

[40] Balabhadra S., Debasu M., Brites C., Nunes L.A.O., Malta O., Rocha J., Bettinelli M., Carlos L. (2015). Boosting the sensitivity of Nd^3+^-based luminescent nanothermometers. Nanoscale.

[41] Sugimoto T., Zhou X., Muramatsu A. (2003). Synthesis of uniform anatase TiO_2_ nanoparticles by gel–sol method. J. Colloid Interface Sci..

[42] Guttmann P., Bittencourt C., Rehbein S., Umek P., Ke X., van Tendeloo G., Ewels C.P., Schneider G. (2011). Nanoscale spectroscopy with polarized X-rays by NEXAFS-TXM. Nat. Photon..

[43] Bittencourt C., Hitchock A.P., Ke X., van Tendeloo G., Ewels C.P., Guttmann P. (2012). X-ray absorption spectroscopy by full-field X-ray microscopy of a thin graphite flake: Imaging and electronic structure via the carbon K-edge. Beilstein J. Nanotechnol..

[44] Colomer M.T., Roa C., Ortiz A.L., Ballesteros L.M., Molina P. (2020). Influence of Nd^3+^ doping on the structure, thermal evolution and photoluminescence properties of nanoparticulate TiO_2_ xerogels. J. Alloy. Compd..

[45] Biesinger M.C., Payne B.P., Grosvenor A.P., Lau L.W., Gerson A.R., Smart R.S. (2011). Resolving surface chemical states in XPS analysis of first row transition metals, oxides and hydroxides: Cr, Mn, Fe, Co and Ni. Appl. Surf. Sci..

[46] Wei Z., Rosa L., Wang K., Endo M., Juodkazis S., Ohtani B., Kowalska E. (2017). Size-controlled gold nanoparticles on octahedral anatase particles as efficient plasmonic photocatalyst. Appl. Catal. B Environ..

[47] Janczarek M., Wei Z., Endo M., Ohtani B., Kowalska E. (2016). Silver- and copper-modified decahedral anatase titania particles as visible light-responsive plasmonic photocatalyst. J. Photon. Energy.

[48] Parnicka P., Mazierski P., Grzyb T., Wei Z., Kowalska E., Ohtani B., Lisowski W., Klimczuk T., Nadolna J. (2017). Preparation and photocatalytic activity of Nd-modified TiO_2_ photocatalysts: Insight into the excitation mechanism under visible light. J. Catal..

[49] Nithyaa N., Jaya N.V. (2021). Effect of Nd on structural, optical and magnetic behaviour of TiO_2_ nanoparticles. Appl. Phys. A.

[50] Thole B.T., van der Laan G., Fuggle J.C., Sawatzky G.A., Karnatak R.C., Esteva J.-M. (1985). *3d* X-ray-absorption lines and the 3*d*94*f*^n+1^ multiplets of the lanthanides. Phys. Rev. B.

[51] Palina N., Wang L., Dash S., Yu X., Breese M.B.H., Wang J., Rusydi A. (2017). Investigation of the metal–insulator transition in Nd NiO_3_ films by site-selective X-ray absorption spectroscopy. Nanoscale.

[52] Pallotti D.K., Passoni L., Maddalena P., Di Fonzo F., Lettieri S. (2017). Photoluminescence mechanisms in anatase and rutile TiO_2_. J. Phys. Chem. C.

[53] Melnyk V., Shymanovska V., Puchkovska G., Bezrodna T., Klishevich G. (2005). Low-temperature luminescence of different TiO_2_ modifications. J. Mol. Struct..

[54] Luo W., Li R., Chen X. (2009). Host-sensitized luminescence of Nd^3+^ and Sm^3+^ ions incorporated in anatase titania nanocrystals. J. Phys. Chem. C.

[55] Ghigna P., Speghini A., Bettinelli M. (2007). Unusual Ln^3+^ substitutional defects: The local chemical environment of Pr^3+^ and Nd^3+^ in nanocrystalline TiO_2_ by Ln–K edge EXAFS. J. Solid State Chem..

[56] Rocha U., da Silva C.J., Silva W.F., Guedes I., Benayas A., Maestro L.M., Elias M.A.A., Bovero E., van Veggel F.C.J.M., Solé J.A.G. (2013). Subtissue thermal sensing based on neodymium doped LaF_3_ nanoparticles. ACS Nano.

[57] Bednarkiewicz A., Marciniak L., Carlos L.D., Jaque D. (2020). Standardizing luminescence nanothermometry for biomedical applications. Nanoscale.

