# Supplementary material for: Nd3+-Doped TiO2 Nanoparticles as Nanothermometer: High Sensitivity in Temperature Evaluation inside Biological Windows
Source: Sensors (Basel). 2021 Aug 6;21(16):5306. doi: 10.3390/s21165306 (PMC8399183; doi:10.3390/s21165306)
Supplement: Supplementary file 1 [file sensors-21-05306-s001.zip › sensors-1271201-supplementary.pdf]

Supporting information

Nd^{3+} doped TiO_2 nanoparticles as nanothermometer: high sensitivity in temperature evaluation inside biological windows

Selene Acosta[1,2], Luis J. Borrero-González[3], Polona Umek[4], Luiz A.O. Nunes[5], Peter Guttmann[6] and Carla Bittencourt[1]

[1]Chimie des Interactions Plasma–Surface (ChIPS), Research Institute for Materials Science and Engineering, Université de Mons, 7000 Mons, Belgium.

[2]Centro de Investigación en Ciencias de la Salud y Biomedicina, Universidad Autónoma de San Luis Potosí., San Luis Potosí 78210, México.

[3]Facultad de Ciencias Exactas y Naturales, Escuela de Ciencias Físicas y Matemática, Pontificia Universidad Católica del Ecuador, Av. 12 de octubre 1076, Apartado, 17-01-2184, Quito, Ecuador.

[4]Jožef Stefan Institute, Jamova cesta 39, 1000 Ljubljana, Slovenia.

[5]Instituto de Física de São Carlos, Universidade de São Paulo, 13566-590 São Carlos, SP, Brazil.

[6] Helmholtz-Zentrum Berlin für Materialien und Energie GmbH, Department X-Ray microscopy, Wilhelm-Conrad-Röntgen Campus, 12489 Berlin, Germany.

Table S1. Summarized data for phase composition, neodymium content and band gap for undoped and Nd^{3+}-doped TiO_2 samples.

**Sample's label**	**Phase composition**	**Nd content / wt. %***	**Band gap / eV**
**TiO_2**	anatase, rutile	/	**3.1** (3.11)
**0.5-Nd-TiO_2**	anatase	0.6 wt%±0.3	**3.0** (3.04)
**1-Nd-TiO_2**	anatase	0.9 wt%±0.2	**3.1** (3.11)
**3-Nd-TiO_2**	anatase	2.9 w%±0.4	**3.00** (2.99)

*Nd content was determined with EDS in combination with SEM

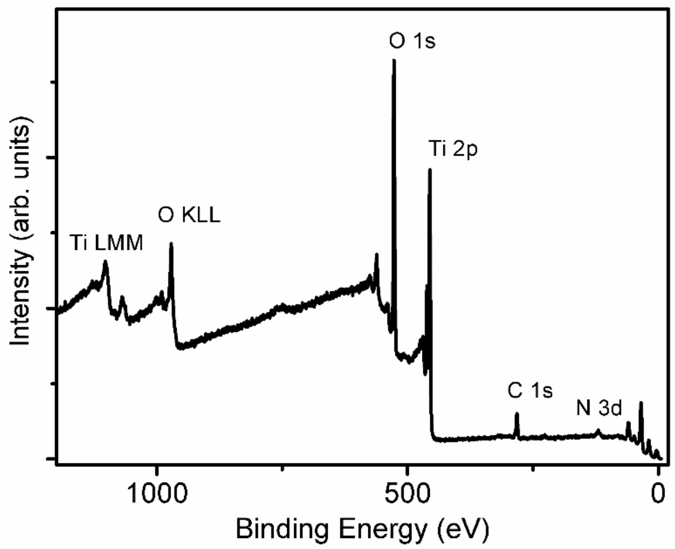

Figure S1: XPS survey spectrum recorded on 3-Nd-TiO_2 sample.

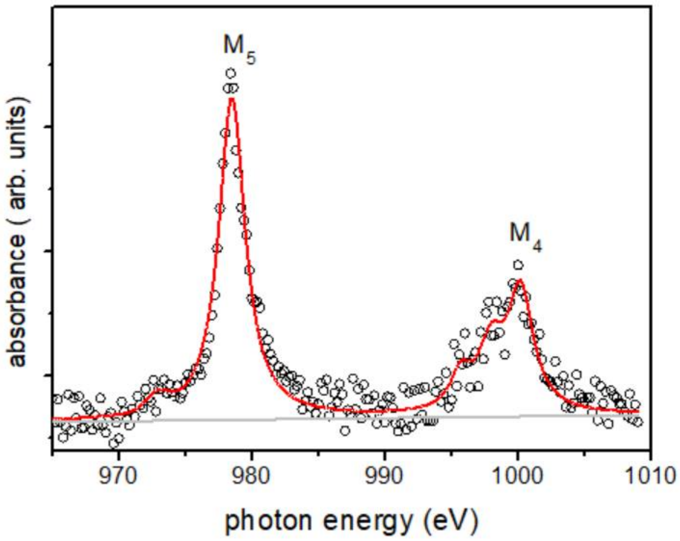

Figure S2. M-edges spectrum recorded on the 0.5-Nd-TiO_2 sample. The horizontal axe gives the excitation photon energy in eV. The open circles are the experimental data and the red line a guide to eye.

The photoluminescence spectra of the samples excited with 350 nm are showed in **Figure S3**. All these spectra have a broad band ranging from 380 nm to 750 nm. The broad band corresponds to emissions from the defect band in TiO_2 due to the self-trapped excitons (STE) luminescence in TiO_2 (**Figure 4**).

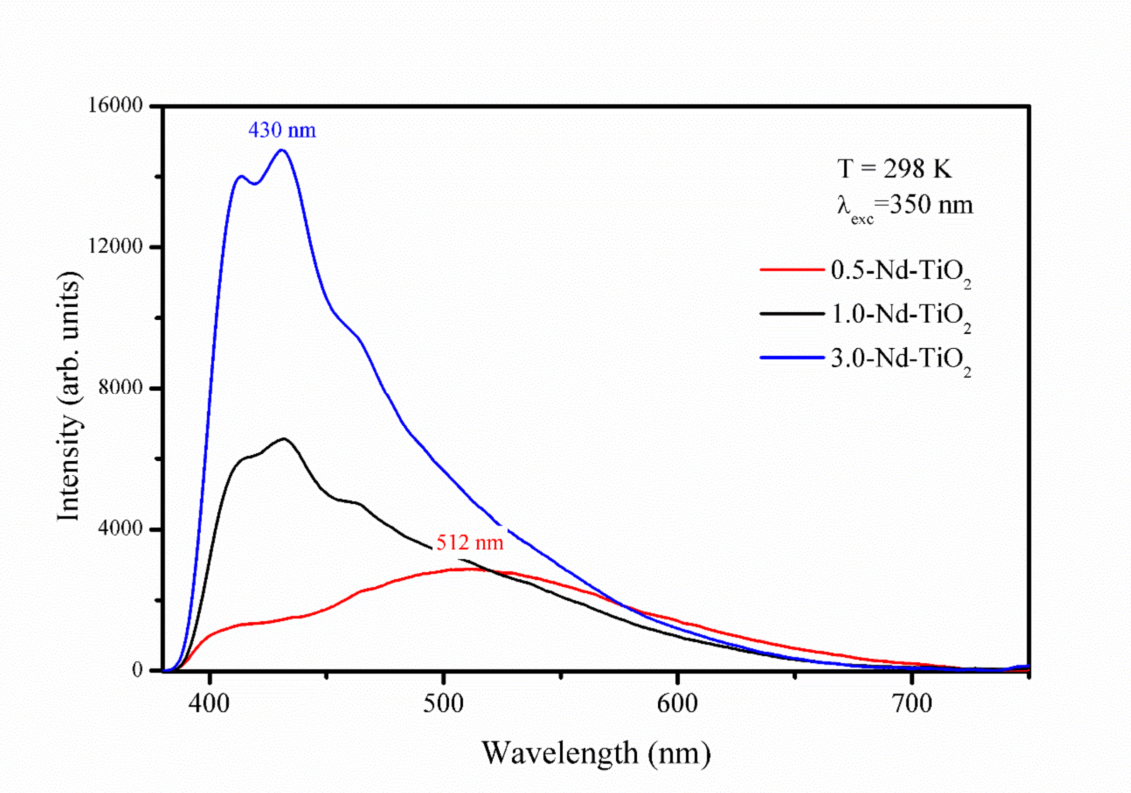

Figure S3. Luminescence spectra of *x*-Nd-TiO_2 (*x*=0.5, 1.0 and 3.0 wt%) nanoparticles, excitation at 350 nm and temperature of 298 K.

The normalized photoluminescence spectra of the samples excited with 514 nm are showed in **Figure S4**. The spectra were corrected to the Nd^{3+} concentration. The emissions at 917 nm, 1096 nm and 1381 nm are due to the Nd^{3+}:$^4F_{3/2} \rightarrow {}^4I_{9/2}$, $^4F_{3/2} \rightarrow {}^4I_{11/2}$ and $^4F_{3/2} \rightarrow {}^4I_{13/2}$ transitions. Pulsed laser excitation at 532 nm was used to estimate the average decay times (τ) and are shown in the legend.

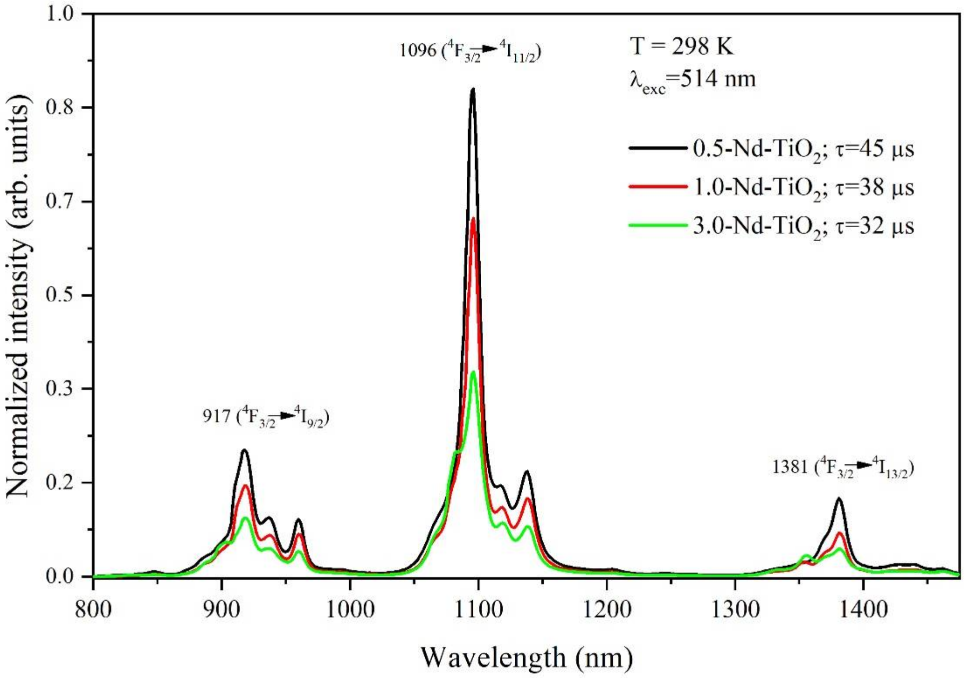

Figure S4. Normalized infrared photoluminescence spectra of *x*-Nd-TiO_2 (*x*=0.5, 1.0 and 3.0 wt%) under 514 nm excitation. The spectra were corrected to the Nd^{3+} concentration. The average decay times (τ) of each sample are shown in the legend.
